# Use of the Health Belief Model for the Assessment of Public Knowledge and Household Preventive Practices in Karachi, Pakistan, a Dengue-Endemic City

**DOI:** 10.1371/journal.pntd.0005129

**Published:** 2016-11-10

**Authors:** Taranum Ruba Siddiqui, Saima Ghazal, Safia Bibi, Waquaruddin Ahmed, Shaimuna Fareeha Sajjad

**Affiliations:** 1 Pakistan Health Research Council, PHRC Research Centre, Jinnah Postgraduate Medical Centre, Karachi, Sindh, Pakistan; 2 Field Epidemiology & Laboratory Training Program (FELTP), Islamabad, Pakistan; Oxford University Clinical Research Unit, VIET NAM

## Abstract

**Background:**

Prevention is most effective in reducing dengue infection risk, especially in endemic countries like Pakistan. Evaluation of public awareness and health beliefs regarding dengue fever (DF) is important for devising disease control strategies. This study assessed dengue knowledge, health beliefs, and preventive practices against DF in different socioeconomic groups of Karachi, Pakistan.

**Methodology:**

In this community-based cross-sectional study, 6 randomly selected towns were visited, 2 persons (man and woman) per household were interviewed using a structured questionnaire, and household practices were observed. Information regarding DF was shared through a printed pamphlet. Multivariate logistic regression analysis of variables associated with dengue knowledge and practices was conducted.

**Principal Findings:**

We interviewed 608 Karachi residents (mean age: 33.2 ± 13.35 years); 7.7%, 71.9%, and 20.4% had a high, middle, and low socioeconomic status, respectively. The mean knowledge score was 6.4 ± 2.10 out of 14. The mean preventive practices score was 9 ± 1.8 out of 17. Predictors of dengue knowledge were perceived threat (odds ratio [OR] = 1.802; 95% confidence interval [CI] = 1.19–2.71; p = 0.005), self-efficacy (OR = 2.910; 95% CI = 1.77–4.76; p = 0.000), and television as an information source (OR = 3.202; 95% CI = 1.97–5.17; p = 0.000). Predictors of dengue preventive practices were perceived threat (OR = 1.502; 95% CI = 1.02–2.19; p = 0.036), self-efficacy (OR = 1.982; 95% CI = 1.34–2.91; p = 0.000), and dengue knowledge (OR = 1.581; 95% CI = 1.05–2.37; p = 0.028).

**Conclusions:**

Public knowledge about DF is low in Karachi. Knowledge, threat perception, and self-efficacy are significant predictors of adequate dengue preventive practices. Prevention and control strategies should focus on raising awareness about dengue contraction risk and severity through television. Health messages should be designed to increase individual self-efficacy.

## Author Summary

Dengue is a massive health threat worldwide. Dengue prevention is the most effective way to reduce the risk of dengue infection, especially in endemic countries like Pakistan. Evaluation of public awareness and health beliefs regarding dengue fever plays an important role in developing strategies for disease control. The present study results highlight the public awareness level, health beliefs, and actual preventive practices regarding dengue fever. We evaluated dengue knowledge and preventive practices in relation with demographic characteristics and public health beliefs. Our findings are useful for developing health messages regarding dengue prevention and control. We found that public awareness has a great impact on adoption of dengue preventive practices. We also found that dengue information should be disseminated in the population in order to increase the perception of susceptibility to contracting this disease. It is also important that health messages include information that augments public confidence that implementation of proper preventive practices can help avoid this infection.

## Introduction

Dengue fever is a massive health threat throughout the world, with the global estimate of the dengue-affected population reaching almost 400 million [1]. Factors augmenting dengue spread are uncontrolled urbanization, population growth, and lack of preventive measures in endemic areas [2]. A local study reported an incidence rate of 570/100,000 per year in the 10 to 15 years age group [3]. In 2011, 22,562 dengue cases were confirmed, with 363 deaths recorded in the country. In the Sindh province alone, 952 cases were reported, with 18 deaths, of which 755 cases, including 15 deaths, were from the Karachi metropolis alone [4]. In 2015, 3,212 cases were detected in Karachi, with an incidence rate of 35.6 per 100,000 in the 9-million population. Many additional cases are underreported or missed due to mild symptoms and inappropriate surveillance for dengue [5].

Dengue fever is a severe influenza-like infection that affects all age groups and rarely causes death, but in developing countries like Pakistan, dengue has the potential to cause high mortality because of an improper water system and sanitation, a large number of refugees, uncontrolled urbanization, improper urban infrastructure, frequent natural disasters, and a lack of resources [6]. The World Health Organization has been working with the Ministry of Health of Pakistan to cope with the situation. This includes epidemiological work on the dengue vector, the *Aedes* mosquito, in Karachi in 2005, followed by the design of a Pakistan-specific control intervention plan. In 2008, the plan was merged with the malaria control program to provide effective, long-term control and prevention of dengue. Despite these efforts, the incidence of dengue is still rising in many parts of the country [7].

Dengue prevention and control can be achieved by adopting proper preventive measures such as the use of mosquito nets, repellent spray, and mosquito coils; removal of stagnant water; and avoidance of trash accumulation in and outside houses to prevent mosquito breeding. In this regard, community awareness about dengue, its perceived severity and susceptibility, and practices and beliefs have a great impact on the prevention and control of dengue in the community [8].

There are factors like socioeconomic status, gender, literacy, and household income that influence the awareness/knowledge, beliefs, and preventive health behavior of an individual [9]. It is important to identify the influence of these factors according to the target population to form an effective plan for the control and prevention of any disease. Dengue knowledge, prevention practices, and the associated demographic factors in relation to Health Belief Model (HBM) constructs have never been explored in the population of the Karachi metropolis, Pakistan. In this study, using the HBM constructs, we attempted to find out individuals’ perceived dengue threat and their household practices for the prevention and control of dengue. The HBM constructs can be used to predict why people take action to control or prevent a particular illness or disease. These constructs are perceived threat of a particular condition, perceived benefits and barriers, perceived self-efficacy (ability to avoid dengue through preventive practices), and cues to action (measures that may increase awareness and readiness in executing preventive practices) [10]. These factors could guide the design of dengue-related targeted interventions and the development of an effective educational/awareness program for the targeted population.

## Methods

This was a cross-sectional community-based study. Out of the 18 towns of Karachi, 6 towns (Gulberg Town, Liaquatabad Town, Saddar Town, Jamshed Town, Bin Qasim Town, and Kiamari Town) were selected for data collection, using the lottery method. Each town is divided into a different number of areas. Through lottery, one randomly selected area was chosen from each town: Aisha Manzil (Gulberg Town), Liaquatabad (Liaquatabad Town), Clifton (Saddar Town), Pakistan Employees Co-operative Housing Society (Jamshed Town), Cattle colony (Bin Qasim Town), and Maripur (Kiamari Town). For each district, a list of mohala/sectors was acquired from the respective Union Council offices. Five sectors were chosen through lottery, and 20 houses from each selected sector were selected systematically (every third house) for data collection. Visits for data collection from systematically selected houses were done spontaneously, with respondents not expecting the visit. The data collection team comprised 4 men and 2 women. This study was approved by the institutional Ethics Review Committee of Jinnah Postgraduate Medical Centre, Karachi.

The selection of participants was based on 1) participation in home chores and responsibility for making decisions concerning the health of the family, 2) age of 18 years or above, 3) provision of written informed consent for participation in the study, and 4) residence in the respective town for more than 5 years. According to the estimated sample size, a total of 608 respondents were recruited for this study. After obtaining verbal and written informed consent, 1 man and 1 woman from each house were interviewed using a structured pretested questionnaire. Household practices and cleanliness of the home were also observed and recorded.

There were a total of 35 questions. The questionnaire was based on a literature review. It was validated by 6 experts who judged every item on the basis of its relevance and clarity. Each item was graded as “not relevant,” “somewhat relevant,” “quite relevant,” or “highly relevant.” The content validity index for the 35 items was calculated to be 0.89. The questionnaire was pretested in the population.

The questionnaire explored sociodemographic information, knowledge about dengue, experience of dengue, perceived threat of dengue, participant opinion on or understanding of the ability to effectively take measures to prevent dengue, i.e., self-efficacy, and observation of practices related to dengue prevention and control.

The following sociodemographic information was obtained from the participants: 1) age, 2) gender, 3) education, 4) socioeconomic status, 5) approximate household income, and 6) number of dependents. The socioeconomic status was determined on the basis of household income per person. A per person monthly income of <3,000, 3,000–5,000, and >5000 rupees (PKRs) was considered to indicate a low, middle, and high socioeconomic status, respectively.

Knowledge about dengue was evaluated with questions on 1) dengue vector, 2) transmission of dengue, 3) dengue signs and symptoms, 4) places where dengue mosquitos can breed and measures to avoid breeding, and 5) treatment and preventive measures to avoid mosquito bites. All correct answers were scored as 1, and incorrect answers and responses indicating uncertainty were scored as 0. If a question had more than one correct answer, each of them was scored as 1. The total score for the questions regarding knowledge about dengue was 14. Knowledge about dengue was considered poor if the respondent scored ≤7. A knowledge score between 8 and 14 was considered good.

Participants’ understanding of/feeling of vulnerability to contracting dengue fever, called perceived susceptibility, and its seriousness, including health consequences, i.e., perceived severity, were evaluated together as perceived threat. This was scored from 0 to 9, with scores of 0 to 5 considered low and scores of 6 to 9 considered high for the purpose of the present analysis.

In the present study, self-efficacy was measured on the basis of 3 activity questions. These questions were designed to assess the level of self-efficacy, i.e., the number of activities an individual thought could be performed to avoid dengue infection. Participants who responded “Yes” for all activities were considered as confident to take measures to prevent dengue infection.

Practices regarding dengue prevention and control were assessed by asking questions as well as by observation of household practices. The questions regarding practices were about the use of mosquito repellent, changing of water in flowerpots, the use of mosquito nets, and the removal of stagnant water from the roof as well as from any inside areas of the house. Observations were made regarding the cleanliness of the home; whether water containers were covered; and the presence of stagnant water near plants, in used bottle piles, in pets’ water containers, as well as in bathrooms. Each positive practice was scored as 1, with a maximum possible total practice score of 17. A score ≤9 was considered to indicate inadequate practices, and a score from 10 to 17 was considered to indicate adequate practices.

After the interview and data collection, a dengue information pamphlet written in the native language was distributed among the respondents. In addition, the respondents were educated about dengue, preventive practices, and the individual’s role in combatting this infection through verbal communication.

Data analysis was done on Microsoft Office Excel, using Statistical Package for Social Science, version 21. The chi-square test was done to identify significant associations of dependent variables (dengue knowledge and preventive practices) with gender, age, literacy, socioeconomic status, monthly income, perceived threat, and self-efficacy. For all statistical analyses, a p-value less than 0.05 was considered to indicate statistical significance. The association between sources of information and dengue knowledge, preventive practices, perceived threat of dengue, and self-efficacy was also analyzed. Logistic regression analysis was done to define the partial contribution of each independent variable to dependent variables like preventive practices score and knowledge score. Variables with a p-value less than 0.05 were included in logistic regression analysis. Variation in the dependent variable was assessed using Cox-Snell R-square. Nagelkerke’s R-square was also used to measure the relationship between predictors and predictions. Model chi-square and Hosmer-Lemeshow tests were also used to check the goodness of model fit.

## Results

### Participant Characteristics and Demographics

In total, 311 households from all the 6 selected towns were approached. Seven (2.3%) households refused to participate in the study. Responses of a total of 608 participants were recorded. The mean age of study participants was 33.2 (±13.35) years; 302 (49.6%) were male and 306 (50.3%) were female.

Out of the 302 male respondents, 158 (52.3%) were reported to be the head of the household. A total of 68 (22.2%) female respondents out of 306 were elderly women; the remaining women were involved in household chores. There were 231 (76%) male and 228 (74%) female literate respondents. Overall, out of these 608 participants, 47 (7.7%), 437 (71.9%), and 124 (20.4%) belonged to the high, middle, and low socioeconomic status groups, respectively. Among these respondents, 149 (24.5%) were illiterate or completed less than 10 grades, whereas 459 (75.5%) completed 10 or more grades and were considered literate. A total of 417 (68.6%) reported a household monthly income of PKRs 20,000 or less, and 191 (31.4%) reported a household monthly income of more than PKRs 20,000. Analysis of associations of the sources of dengue information with dengue knowledge, preventive practices, perceived threat of dengue, and self-efficacy showed a significant association for television, newspapers, and the government campaign performed to promote dengue knowledge in the community (Table 1). The government campaign was the “Dengue Prevention and Control Program,” which has been launched to provide surveillance data, spray insecticides, and improve public knowledge to implement adequate dengue preventive practices.

**Table 1:** Associations of Information Sources with Dengue Knowledge, Preventive Practices, Perceived Threat, and Self-Efficacy.

Sources of Dengue Information	Dengue Knowledge Score		Preventive Practices Score		Perceived Threat Score		Self-Efficacy Score	
	≤7	8–14	≤9	10–17	≤5	6–9	1–2	3
**Television**								
**Yes**	246 (59) ^a^	168 (41)	153 (37)	261 (63)	180 (44)	234 (56)	112 (27)	300 (73)
**No**	165 (85)	29 (15)	92 (47)	102 (53)	121 (64)	68 (36)	74 (38)	119 (62)
**P-value**	**0.000** ^b^		**0.018**		**0.000**		**0.007**	
**Radio**								
**Yes**	15 (60)	10 (40)	10 (40)	15 (60)	5 (21)	19 (79)	8 (32)	17 (68)
**No**	396 (68)	187 (32)	235 (40)	348 (60)	296 (51)	283 (49)	178 (31)	402 (69)
**P-value**	0.541		1.000		**0.007**		1.000	
**Newspapers**								
**Yes**	59 (58)	43 (42)	29 (28)	73 (72)	20 (20)	82 (80)	19 (19)	83 (81)
**No**	352 (70)	154 (30)	216 (43)	290 (57)	281 (56)	220 (44)	167 (33)	336 (67)
**P-value**	**0.028**		**0.010**		**0.000**		**0.005**	
**Magazines**								
**Yes**	19 (63)	11 (37)	11 (37)	19 (63)	5 (17)	25 (83)	9 (30)	21 (70)
**No**	392 (68)	186 (32)	234 (41)	344 (59)	296 (52)	277 (48)	177 (31)	398 (69)
**P-value**	0.755		0.822		**0.000**		1.000	
**Books**								
**Yes**	31 (65)	17 (35)	17 (35)	31 (65)	8 (17)	40 (83)	10 (21)	38 (79)
**No**	380 (68)	180 (32)	228 (41)	332 (59)	293 (53)	262 (47)	176 (32)	381 (68)
**P-value**	0.761		0.572		**0.000**		0.165	
**Friends**								
**Yes**	56 (64)	32 (36)	20 (28)	52 (72)	27 (31)	60 (69)	20 (23)	68 (77)
**No**	355 (68)	165 (32)	225 (42)	311 (58)	274 (53)	242 (47)	166 (32)	351 (68)
**P-value**	0.462		0.161		**0.000**		0.101	
**Family**								
**Yes**	47 (65)	25 (35)	20 (28)	52 (72)	18 (25)	54 (75)	16 (22)	56 (78)
**No**	364 (68)	172 (32)	225 (42)	311 (58)	283 (53)	248 (47)	170 (32)	363 (68)
**P-value**	0.753		**0.029**		**0.000**		0.125	
**Government campaign**								
**Yes**	41 (50)	41 (50)	18 (22)	64 (78)	20 (24)	62 (76)	14 (17)	68 (83)
**No**	370 (70)	156 (30)	227 (43)	299 (57)	281 (54)	240 (46)	172 (33)	351 (67)
**P-value**	**0.000**		**0.000**		**0.000**		**0.006**	

^a^ Values are presented as number (percentage).

^b^ Bold font indicates statistical significance.

### Dengue Knowledge

A total of 522 (85.9%) respondents claimed that they knew about dengue infection. A total of 517 (85%) knew that it is spread by mosquitos. The main symptoms of dengue were reported as headache by 174 (28.6%), body pain by 284 (46.7%), and eye pain by 80 (13.2%); 440 (72.4%) indicated that dengue infection can occur in all age groups. Only 198 (32.6%) were aware that dengue fever mostly occurs in the monsoon (rainy) season, and 419 (68.9%) reported that dengue mosquitos breed in fresh water. One hundred eighty-six (30.6%), 173 (28.5%), and 240 (39.5%) respondents agreed that used tires, used/spare boxes, and water present in flower pots/vases are sites for mosquito breeding. Almost all the respondents, 586 (96.4%), agreed that water at home should be stored in a covered utensil/tank, and 344 (56.6%) reported that uncovered water utensils should be cleaned on a daily basis. A total of 353 (58.1%) stated that this is a contagious infection that can be transmitted from one person to another.

**Fig 1:**
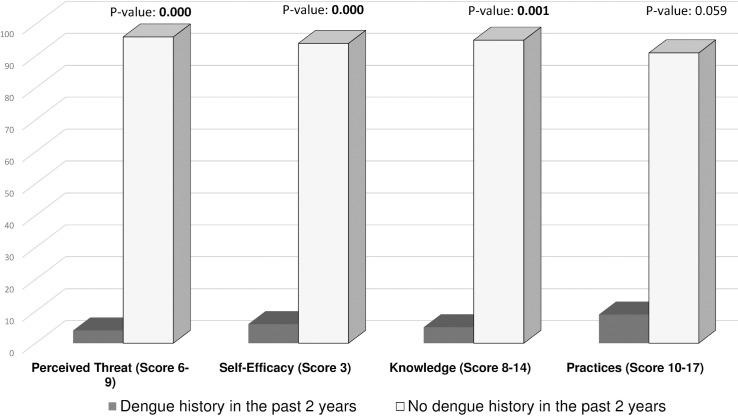
History of Dengue Fever in the Past 2 Years and Its Association with Dengue Knowledge, Practices, and Health Beliefs. Bars represent percentages of respondents with high perceived threat of dengue, self-efficacy, adequate dengue knowledge, and preventive practices scores according to the history of dengue fever in the past 2 years. P-values shown in bold indicate a significant association.

**Fig 2:**
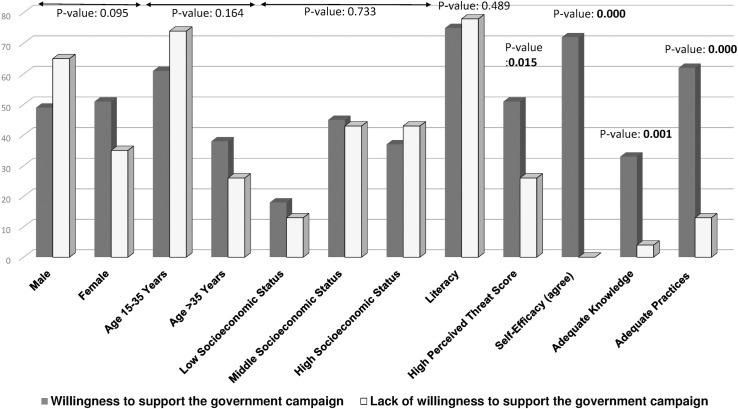
Willingness to Support the Government Campaign and Its Association with Demographics, Dengue Knowledge, Practices, and Health Beliefs. Bars represent the willingness to support the government campaign for dengue prevention according to demographic characteristics, perceived threat of dengue, self-efficacy, and preventive practices. P-values shown in bold indicate a significant association.

The mean score for knowledge about dengue fever was 6.4 ± 2.10 out of 14. As shown in Table 2, there were significant associations between dengue knowledge and age, literacy, history of having dengue fever in the last 2 years, perceived threat, and self-efficacy. We found it interesting that those who had suffered from dengue fever in the past 2 years had a significantly lower knowledge score. A further, separate analysis of a past dengue history with perceived threat of dengue, self-efficacy, and practices revealed that perceived threat and self-efficacy were significantly associated with a history of dengue fever (p < 0.01) (Fig 1). When we compared the respondents’ willingness to participate in the government campaign with health beliefs, knowledge, and practices, a significant association (p < 0.05) was found with these variables (Fig 2).

**Table 2:** Univariate Analysis of the Association of Knowledge about Dengue with Demographic and Other Determinants (N = 608).

Sociodemographic Variables	Frequency	Dengue Knowledge		P-Value	Odds Ratio	95% Confidence Limit
		Inadequate	Adequate			
		Score: ≤7	Score: 8–14			
**Age (years)**						
15 to 35	377 (62.0) ^a^	267 (70.8)	110 (29.2)	**0.037** ^b^	1.466	1.03–2.07
36 to 55	231 (38.0)	144 (62.3)	87 (37.7)			
**Literacy**						
Illiterate	149 (24.5)	113 (75.8)	36 (24.2)	**0.018**	1.696	1.11–2.58
Literate	459 (75.5)	298 (64.9)	161 (35.1)			
**Dengue fever in the last 2 years**						
Yes	66 (10.9)	56 (84.8)	10 (15.2)	**0.002**	2.950	1.47–5.91
No	542 (89.1)	355 (65.5)	187 (34.5)			
**Perceived threat score**						
0 to 5	301 (49.9)	237 (78.7)	64 (21.3)	**0.000**	2.914	2.03–4.16
6 to 9	302 (50.1)	169 (56.0)	133 (44.0)			
**Self-efficacy**						
Disagree	186 (30.7)	160 (86.0)	26 (14.0)	**0.000**	4.243	2.68–6.70
Agree	419 (69.3)	248 (59.2)	171 (40.8)			
**Dengue preventive practices scores**						
≤9	245 (40.3)	192 (78.4)	53 (21.6)	**0.000**	2.382	1.64–3.44
10–17	363 (59.7)	219 (60.3)	144 (39.7)			

^a^ Values are presented as number (percentage).

^b^ Bold font indicates statistical significance.

Before regression analysis, its assumptions were verified. The Durbin-Watson test statistic was used for verification of the independence of error assumption. The value of the Durbin-Watson test statistic was within the acceptable range (1.434 and 1.511). Thus, we failed to reject the null hypothesis that there was no correlation among residuals, i.e., they were independent.

In multiple logistic regression analysis, the model chi-square was statistically significant (chi-square = 104.77, p < 0.000 with df = 9). The Wald criterion demonstrated that only perceived threat, agreement on self-efficacy, and television as a source of dengue information made a significant contribution to the prediction of high dengue knowledge (Table 3). Self-efficacy (odds ratio [OR] = 2.910; 95% confidence interval [CI] = 1.77–4.76; p = 0.000) and television (OR = 3.202; 95% CI = 1.97–5.17; p = 0.000) as a source of information were associated with higher (3-fold) odds of adequate dengue knowledge. Higher perceived threat scores (OR = 1.802; 95% CI = 1.19–2.71; p = 0.005) were associated with higher (2-fold) odds of adequate dengue knowledge. Age, literacy, history of dengue fever in the last 2 years, and sources of information, i.e., newspapers and the government campaign, were not significant predictors of dengue knowledge.

**Table 3:** Multivariate Logistic Regression Analysis of Variables Associated with Dengue Knowledge.

Sociodemographic Variables	Logistic Regression Model of Poor vs. Good Dengue Knowledge		
	P-value	Adjusted odds ratio	95% confidence limit
**Age (years)**			
15 to 35	0.219	Reference	
36 to 55		1.271	0.86–1.86
**Literacy**			
Illiterate	0.551	Reference	
Literate		1.155	0.71–1.85
**Dengue fever in the last 2 years**			
Yes	0.920	Reference	
No		0.959	0.42–2.16
**Perceived threat score**			
0 to 5	**0.005** ^a^	Reference	
6 to 9		1.802	1.19–2.71
**Self-efficacy score**			
Disagree	**0.000**	Reference	
Agree		2.910	1.77–4.76
**Dengue preventive practices score**			
≤9	**0.026**	Reference	
10–17		1.587	1.05–2.38
**Information source (television)**			
Yes	**0.000**	3.202	1.97–5.17
No		Reference	
**Information source (newspapers)**			
Yes	0.960	1.013	0.61–1.66
No		Reference	
**Information source (the government campaign)**			
Yes	0.118	1.514	0.90–2.54
No		Reference	

^a^ Bold font indicates statistical significance.

### Dengue Preventive Practices

Regarding dengue practices, 292 (48%), 230 (37.8%), 138 (22.7%), and 109 (17.9%) reported that mosquito eradication, use of mosquito nets, body covering with cloths, and application of mosquito repellent lotions were effective in avoiding mosquito bites. Most of the respondents, 543 (89.3%), covered water storage utensils. Further, 399 (65.6%) respondents stated that they changed flower pots/vases daily, only 125 (20.6%) used mosquito nets, 320 (52.6%) used coils, 148 (24.3%) used mosquito repellent, 152 (25%) used mosquito sprays at home, and a few of them, 44 (7.2%), also used smoke to avoid mosquito bites. Household observation showed that the houses of 572 (94.1%) respondents were clean. Flower pots and plants were clean in the houses of 489 (80.4%) respondents, uncovered water pots were seen in the houses of 176 (28.9%) respondents, and 189 (31.1%) respondents had empty pots and boxes in which water could be stored and could serve as a place for mosquito breeding. Three hundred twenty (52.6%) respondents had clean uncovered pet water containers. The mean score of preventive practices was 9 ± 1.8 out of 17. Dengue preventive practices were significantly associated with age, literacy, perceived threat, self-efficacy, and dengue knowledge (Table 4). Regarding sources of dengue information, we found that television, newspapers, family, as well as the government campaign were significantly associated with dengue preventive practices (Table 1).

**Table 4:** Univariate Analysis of the Association of Dengue Preventive Practices with Demographic and Other Determinants (N = 608).

Sociodemographic Variables	Frequency	Dengue Preventive Practices		P-Value	Odds Ratio	95% Confidence Limit
		Inadequate	Adequate			
		Score: ≤9	Score: 10–17			
**Age (years)**						
15 to 35	377 (62.0) ^a^	165 (43.8)	212 (56.2)	**0.032** ^b^	1.469	1.04–2.06
36 to 55	231 (38.0)	80 (34.6)	151 (65.4)			
**Literacy**						
Illiterate	149 (24.5)	72 (48.3)	77 (51.7)	**0.028**	1.546	1.06–2.24
Literate	459 (75.5)	173 (37.7)	286 (62.3)			
**Perceived threat score**						
0 to 5	301 (49.9)	151 (50.2)	150 (49.8)	**0.000**	2.334	1.67–3.25
6 to 9	302 (50.1)	91 (30.1)	211 (69.9)			
**Self-efficacy**						
Disagree	186 (30.7)	105 (56.5)	81 (43.5)	**0.000**	2.668	1.87–3.80
Agree	419 (69.3)	137 (32.7)	282 (67.3)			
**Knowledge score**						
≤7	411 (67.6)	192 (46.7)	219 (53.3)	**0.000**	2.382	1.64–3.44
8–14	197 (32.7)	53 (26.9)	144 (73.1)			

^a^ Values are presented as number (percentage).

^b^ Bold font indicates statistical significance.

In logistic regression analysis, the model chi-square was statistically significant (chi square = 61.98, p < 0.000 with df = 9), indicating an overall significant model. The Wald criterion indicated that only perceived threat, self-efficacy, and dengue knowledge significantly contributed as true predictors of dengue preventive practices (Table 5). It was observed that higher scores for perceived threat (OR = 1.502; 95% CI = 1.02–2.19; p = 0.036) were associated with higher (2-fold) odds of dengue preventive practices. Similarly, participants with higher knowledge (OR = 1.581; 95% CI = 1.05–2.37; p = 0.028) and self-efficacy (OR = 1.982; 95% CI = 1.34–2.91; p = 0.000) scores had higher (2-fold) odds of implementing dengue preventive practices. Other candidate factors like age, literacy, and sources of information were not significant predictors for adequate dengue preventive practices.

**Table 5:** Multivariate Logistic Regression Analysis of Variables Associated with Dengue Preventive Practices.

Sociodemographic variables	Logistic Regression Model of Inadequate vs. Adequate Dengue Preventive Practices		
	P-value	Adjusted odds ratio	95% confidence limit
**Age (years)**			
15 to 35	0.105	Reference	
36 to 55		1.349	0.93–1.93
**Literacy**			
Illiterate	0.674	Reference	
Literate		1.092	0.72–1.64
**Perceived threat score**			
0 to 5	**0.036** ^**a**^	Reference	
6 to 9		1.502	1.02–2.19
**Self-efficacy**			
Disagree	**0.000**	Reference	
Agree		1.982	1.34–2.91
**Knowledge score**			
≤ 7	**0.028**	Reference	
8–14		1.581	1.05–2.37
**Information source (television)**			
Yes	0.513	1.136	0.77–1.66
No		Reference	
**Information source (newspapers)**			
Yes	0.520	1.184	0.70–1.97
No		Reference	
**Information source (family)**			
Yes	0.295	0.728	0.40–1.31
No		Reference	
**Information source (the government campaign)**			
Yes	0.051	1.794	0.99–3.22
No		Reference	

^a^ Bold font indicates statistical significance.

## Discussion

In this study, most of the respondents, 522 (85.9%), believed that they have knowledge about dengue. Further evaluation revealed that in Karachi, an endemic area for dengue, the mean score of dengue knowledge is very low, i.e., 6.4 ± 2.10 out of 14. The rate of adequate dengue knowledge within the community was only 32.4%. This is comparable with the results of a local study published in 2010 [11] but inconsistent with the study conducted in “Wah Cantonment” Punjab province that showed a 54.3% rate of adequate knowledge [12]. A community survey from a neighboring country Nepal reported a 12% rate of adequate knowledge [13]; our findings are quite fair in comparison to this figure but not satisfactory. Our findings showed that, except for age and literacy, participants’ demographic characteristics are not significantly associated with dengue knowledge (Table 2). In past studies conducted in Karachi, literacy as well as socioeconomic status was significantly associated with adequate dengue knowledge [11,14]. We have not found any significant association between socioeconomic status of the respondent and dengue knowledge. This study showed that the respondent’s age has a significant association with dengue knowledge, with 37.7% of the respondents in the older age group, i.e., 36–55 years, having adequate knowledge as compared to 29.2% in the younger age group, i.e., 15–35 years. Similar results have been obtained in the study conducted in Wah Cantonment [12]. The lack of knowledge in the younger age group indicates that the media and sources of information about dengue might not be adequately reaching this age group compared to older individuals, who willingly show interest and seek information. We found that those who had had dengue fever in the past 2 years had a significantly lower level of dengue knowledge, which contradicts a previous study that showed a significant impact of past dengue exposure on adequate dengue knowledge [9]. From this finding, we may surmise that, in our setup, health care providers do not adequately provide useful information about dengue prevention to their patients. Interestingly, our analysis of a history of dengue in the previous 2 years with the HBM constructs of perceived threat and self-efficacy revealed that it was negatively associated with these constructs. We may surmise that such individuals might be at a higher risk of contracting dengue due to their low perceived threat and self-efficacy. Preventive practices for dengue were also associated with a history of dengue fever in the past 2 years, with the data indicating that adequate dengue preventive practices are very high in those who do not have a past history (Fig 1). On regression analysis, none of these demographic factors significantly contributed as an independent predictor of adequate dengue knowledge in our population. We also tried to determine positive associations of willingness to support the government campaign for dengue with public knowledge about dengue, practices, and health beliefs, and a significant association was found for all these variables. Almost all respondents who had high scores for these variables were willing to participate in and support the government campaign for dengue prevention in their area (Fig 2). No association was found between respondents’ willingness and their age, gender, or socioeconomic status.

Electronic and print media are considered to play an important role in the dissemination of information to the public. Regression analysis showed that radio, newspapers, and others sources of information about dengue are not significant predictors compared to television. Regression analysis suggested that television as an information source was associated with higher (3-fold) odds of knowledge about dengue compared to other sources of information. These findings support other local and international studies that identified television as a useful source of information [14,15]. The reason for information delivery via television being a significant predictor of adequate dengue knowledge could be that television is the most popular form of media that appeals to every socioeconomic class, including the literate and the illiterate, and every age group in our part of the world. Our finding suggests that television as a source of information can be used as an effective means to promote dengue awareness among the masses.

Regarding the HBM constructs, we found that higher perceived self-efficacy and threat scores are associated with higher (2–3-fold) odds of adequate dengue knowledge. These findings are in concordance with a recent study carried out in Malaysia [16]. According to the HBM, perceived threat encompasses 2 main HBM constructs, perceived susceptibility and perceived severity. From these findings, we may infer that a person who feels susceptible to dengue and believes that contracting dengue may lead to severe health consequences will consequently be active in seeking more information about dengue in order to prevent this disease. In addition, this feeling of “self-efficacy,” i.e., the realization that “on adopting precautionary measures this disease can be prevented,” supports information-seeking behavior, eventually leading to adequate dengue knowledge. It is interesting that perceived threat was significantly associated (p = 0.000–0.007) with all sources of information used to increase dengue knowledge (Table 1). We could say that this important HBM construct, perceived threat, can easily be comprehended by our population, regardless of the information source used. The situation is different for self-efficacy, as only television and newspapers showed a significant association with this construct (Table 1). Our findings suggest that the information about dengue disseminated in the community should focus on increasing public perceived threat and perceived susceptibility to acquiring this disease, as well as on the understanding of self-efficacy and assurance that this infection can be prevented by effective preventive practices. With reference to the HBM, framing health messages for dengue prevention according to HBM constructs could result in an effective dengue control program in Karachi [17].

Although the HBM is a conceptual guiding framework for health behavior intervention, it has some limitations. For example, the HBM does not account for environmental factors that may prevent an individual from practicing the desired behaviors. For instance, an individual’s determination to adopt dengue preventive practices may be limited by poor infrastructure, bad sanitation, and bad water supply. In addition, the HBM does not consider the emotional component of behavior. Furthermore, “cues to action” cannot be assessed precisely by the HBM, as it is difficult for an individual to remember the cue that prompted the change of behavior.

To the best of our knowledge, this is the first study from Karachi, Pakistan, that was carried out in the community and that recorded the actual practices of dengue prevention being carried out in households of this dengue-endemic metropolis. It was found that adequate dengue preventive practices were adopted in the households of 363 (59.7%) respondents in the study population. This figure is quite impressive when compared with the 32.4% rate of adequate dengue knowledge in the population. Similar differences have been found in a national survey conducted in Malaysia [9]. These differences might be noted, as knowledge was measured using items that were beyond the scope of dengue preventive practices, i.e., the *Aedes* mosquito, dengue symptoms, seasons, and medication. Moreover, some practices might be performed without knowledge about dengue just because of social acceptability or social norms, like covering utensils, keeping the house clean and clutter free, and removing empty boxes for the sake of cleanliness. Our results showed that age and literacy were significantly associated with dengue preventive practices. Adequate dengue prevention practices were used more often in the 36–55 years age group (151 [65.4%]) than in the 15–35 years age group (212 [56.2%]). Similarly, literate respondents were better at adopting adequate dengue prevention practices (286 [62.3%]) than illiterate respondents (77 [51.7%]). In a study conducted in Punjab, literacy had no association with actual prevention practices [12]. The inconsistency in the results might be due to the differences in population, as Karachi is a multilingual city where population characteristics vary more broadly than in other cities of Pakistan.

Regression analysis showed that none of the demographic and other general features of respondents was found to be a significant predictor of adequate dengue preventive practices. The present study showed that perceived threat, self-efficacy, and knowledge about dengue are the only significant predictors of adequate dengue preventive practices (Table 5). Perceived threat is one of the important factors that increase readiness and motivation to take precautionary measures [18]. Our data suggest that individuals who have sufficient perception of susceptibility to dengue and feel the threat of acquiring the disease have higher odds of adopting preventive practices than others. This is in concordance with the study carried out in the Malaysian population [9]. Regression analysis also showed that those who have a perception of self-efficacy have higher odds of adopting adequate dengue practices than those who do not have confidence that they can prevent dengue through effective preventive measures. Self-efficacy is another HBM construct that, in addition to perceived threat and other constructs, encourages an individual to implement preventive practices [19]. It was found that dengue knowledge is also a significant independent predictor of dengue preventive practices. This is not consistent with the results of a study carried out in Lahore, another dengue-endemic city of Pakistan [20], as well as with a study conducted in the Philippines [15]. Our findings are consistent with the outcome of studies carried out in Malaysia and Cuba [9,21]. We may infer from our findings that public health beliefs have a great impact on dengue preventive practices. Knowledge about dengue should be disseminated at a mass level so that preventive practices can be improved in the population.

From Pakistan, this study is one of its kind in that it evaluated public knowledge about dengue and preventive practices by using the HBM. A limitation of the study is that the questionnaire did not include questions regarding perceived benefits and barriers related to dengue prevention. Thus, we were not able to analyze these important factors, which could also be useful to evaluate public perception of dengue prevention. We tried to minimize the biases related to self-reporting of dengue preventive practices through direct observation.

From this study, we may conclude that knowledge about dengue is rather limited in the population of dengue-endemic Karachi, a metropolitan city of Pakistan. The present study showed that adequate dengue knowledge leads to adequate dengue preventive practices. Socioeconomic status and other demographics are not significant predictors of dengue knowledge and practices in the population. Older and literate individuals have more knowledge about dengue and adopt more preventive practices. Health beliefs are a significant predictor of both adequate dengue knowledge and adequate dengue preventive practices in our population. Hence, it is recommended that health messages and awareness campaigns should be formulated on the basis of these health belief constructs. There is a need for mass campaigning on television to disseminate dengue information emphasizing public susceptibility to dengue and the consequences of contracting this disease. The public message should be modified so that it increases individuals’ self-efficacy, such that they can avoid the contraction of dengue by adopting proper preventive practices.

## Supporting Information

S1 Checklist STROBE Checklist. (DOC)
